# Influence of Parenting Practices on Eating Behaviors of Early Adolescents during Independent Eating Occasions: Implications for Obesity Prevention

**DOI:** 10.3390/nu7105431

**Published:** 2015-10-22

**Authors:** Marla Reicks, Jinan Banna, Mary Cluskey, Carolyn Gunther, Nobuko Hongu, Rickelle Richards, Glade Topham, Siew Sun Wong

**Affiliations:** 1 Department of Food Science and Nutrition, University of Minnesota, 1334 Eckles Ave., St. Paul, MN 55108-6099, USA; 2 Department of Human Nutrition, Food and Animal Sciences, Agricultural Sciences 216, University of Hawaii at Manoa, 1955 East-West Rd, Honolulu, HI 96822, USA; jcbanna@hawaii.edu; 3 College of Public Health and Human Sciences, Nutrition, Oregon State University, 101 Milam Hall, Corvallis, OR 97331-3303, USA; cluskeym@oregonstate.edu; 4 Department of Human Sciences, the Ohio State University, 313 Campbell Hall, 1787 Neil Ave, Columbus, OH 43210, USA; gunther.22@osu.edu; 5 Department of Nutritional Sciences, University of Arizona, 406 Shantz Building, 1177 E. 4th Street, Tucson, AZ 85721-0038, USA; hongu@email.arizona.edu; 6 Department of Nutrition, Dietetics & Food Science, Brigham Young University, S233 Eyring Science Center, Provo, UT 84602, USA; rickelle_richards@byu.edu; 7 Department of Human Development and Family Science, Oklahoma State University, 243 Human Sciences, Stillwater, OK 74078, USA; glade.topham@okstate.edu; 8 College of Public Health and Human Sciences, School of Biological and Population Health Sciences, Hallie E. Ford Center for Healthy Children and Families, Extension Family and Community Health Program, Nutrition, Oregon State University, Ballard Hall 105E, Corvallis, OR 97331-3303, USA; SiewSun.Wong@oregonstate.edu

**Keywords:** early adolescents, independent eating occasions, eating behaviors, parenting practices, obesity prevention

## Abstract

Among early adolescents (10–14 years), poor diet quality along with physical inactivity can contribute to an increased risk of obesity and associated biomarkers for chronic disease. Approximately one-third of United States (USA) children in this age group are overweight or obese. Therefore, attention to factors affecting dietary intake as one of the primary contributors to obesity is important. Early adolescents consume foods and beverages during eating occasions that occur with and without parental supervision. Parents may influence eating behaviors of early adolescents during eating occasions when they are present or during independent eating occasions by engaging in practices that affect availability of foods and beverages, and through perceived normative beliefs and expectations for intake. Therefore, the purpose of this article was to describe the influence of parenting practices on eating behaviors in general and when specifically applied to independent eating occasions of early adolescents. This information may be helpful to inform parenting interventions targeting obesity prevention among early adolescents focusing on independent eating occasions.

## 1. Introduction

Eating occasions for many early adolescents (10–14 years) are characterized by poor overall diet quality and overconsumption of energy [1,2]. Fruit and vegetable (FV) [3], and whole grain and fiber consumption is low [4,5], and intake of sodium and calories from added sugars is high [6,7,8,9]. Poor diet quality along with physical inactivity can contribute to an increased risk of obesity, and consequently to risk factors for heart disease and type 2 diabetes [10,11]. Approximately one-third of children (6–11 years) and adolescents (12–19 years) are overweight or obese based on National Health and Nutrition Examination Survey (NHANES) data (2011–2012) [12]. Thus, efforts to improve aspects of diet quality related to energy balance and obesity prevention are especially important.

Early adolescents consume 63%–65% of their daily calories at home [13], making the home and family environment an important target for interventions to improve diet quality and prevent obesity. Parental behaviors forming part of the home and family environmental sphere of influence within the Socio-Ecological Model [14] include practices, such as making healthy foods available, establishing expectations for healthful food consumption, and setting a good example. These practices have been positively associated with overall diet quality of youth [15,16,17,18]. A similar set of parenting practices has been identified regarding influence on youth physical activity behaviors including modeling, providing support for physical activity in the home environment, and establishing rules or expectations for physical activity [19]. A review of physical activity parenting practices for early adolescents was beyond the scope of the current review, which was focused primarily on eating behaviors.

For this review, independent eating occasions are defined as those without parental supervision in which an early adolescent is eating alone or with siblings or peers. This review (1) characterizes early adolescent eating behavior by describing eating occasions with respect to frequency, intake (energy, nutrients, foods and beverages), and eating context (location, time of day, who is present) with an emphasis on independent occasions; (2) describes the influence of parenting practices on eating behaviors in general and as it pertains to independent eating occasions; and (3) suggests future research needs to inform parenting interventions targeting obesity prevention among early adolescents focusing on independent eating occasions.

## 2. Early Adolescent Eating Occasions

Trends that may contribute to poor diet quality and positive energy balance in children and adolescents in the United States (USA) include an increase in number of eating occasions, portion sizes, energy density, snacking frequency, skipping breakfast, and eating meals away from home. The number of eating occasions for children (2–18 years) in the USA was estimated to be 5.1 per day based on NHANES data (2005–2010), which represented an increase of 1.2 occasions from 1977 to 1978 [20]. Over a similar time period, total energy intake increased by 108 kcal/day for children and adolescents [20] and energy density of foods increased among youth 12–19 years [21]. Consumption of three meals per day (*i.e.*, breakfast, lunch and dinner) was reported less often by boys (68%) and girls (49%) 12–19 years *versus* boys (73%) and girls (69%) 6–11 years according to NHANES (2009–2010) data [22]. More older adolescents aged 12–19 years (26%) did not report eating breakfast than younger children and adolescents 6–11 years (13%) and 19% of older adolescents *versus* 13% of younger children/adolescents did not report eating lunch (NHANES 2009–2010) [22].

Although data regarding snacking frequency and intake were available for early adolescents [15,23,24], limited studies reported eating context for snacks (*i.e.*, who was present, where the snack was consumed). Older children and adolescents may be home alone or with peers and/or siblings after school, thus snacks may often be consumed during occasions unsupervised by an adult. An assessment of meal and snack trends among children and adolescents (2–18 years) based on self-defined eating events showed that approximately 1.11 additional snacks were consumed per day from 1977–1978 to 2003–2006 [23]. Snack intake accounted for about 27% of total energy in 2003–2005 for 7–12 year olds, exceeding 500 kcals/day [15]. The number of snacking occasions remained high among children 6–19 years between 2005 and 2010 with about two-thirds of these occasions occurring after lunch [24].

Snacks are made up largely of salty snacks, sweets and sweetened beverages among adolescents [25]. This finding may explain the positive association observed between snacking frequency and total energy intake among a sample of schoolchildren in Boston [26]. Total daily energy intake increased by about 9% with each additional snack consumed, and snacking was associated with lower diet quality as measured by the 2005 Healthy Eating Index [26]. Increases in energy intake and effects on diet quality may be related to intake of sugar-sweetened beverages (SSBs) with snacks. Among children 6–11 years and 12–19 years, those who drank SSBs were more likely to consume salty snacks and calories from snacks than non-SSB consumers [27]. Increases in energy intake from eating occasions which included soft drinks were observed in an assessment of trends in portion sizes of selected foods over 30 years for children 2–18 years [28].

For foods prepared away from home, the largest contributor to energy intake for children 2–18 years was fast food, providing 13% of total intake (NHANES 2003–2006) [13]. Other away from home food sources, such as schools, full-service restaurants, and vending machines contributed to smaller percentages of calories consumed. Schools and child-care centers contributed about 32% of the intake of calories from solid fat and added sugars for children 2–18 years and 20% of the intake of high-fat milk and pizza (NHANES 2009–2010) [29]. Parents may be with early adolescents during eating occasions at restaurants but unlikely to be present during eating occasions at school. Because breakfast and lunch are offered to most early adolescents in schools, this is an important independent eating occasion setting. Adolescents make decisions in schools about whether to eat breakfast and lunch and which foods to select. These decisions may be influenced by parental expectations and role modeling, whereas availability of foods and food/beverage selection is dependent on the eating environment (foods offered in the cafeteria or vending machines on campus, off campus sources, or food brought from home). For example, the availability of a la carte and vending machine options in schools was inversely associated with intake of FV consumption among 7th grade students [30]. Availability of SSBs in schools was positively associated with SSB intake by students in grades 7–12 in schools [31].

There have been no significant changes in average daily total time spent on eating for adults in America (1.18 hours/day in 2004 *vs.* 1.11 hours/day in 2014) [32]. However, from 2004 to 2014, the frequency of primary eating decreased among adults (eating/drinking without engaging in another activity, more typical of eating meals) while the frequency of secondary eating increased (eating/drinking while engaging in another activity) [32]. Engaging in other activities while eating may lead to “mindless” eating and increased energy intake compared to primary eating [33], which may be important for early adolescents who snack while watching TV or playing video games. However, data on frequency and intake during primary *versus* secondary eating/drinking occasions for early adolescents were not available.

The relationships between meal frequency, snacking, energy intake and body mass index (BMI) remain unclear in adolescents [34,35]. Total amount of food consumed, specifically from snacks, was positively associated with overweight status [36]. However, a review of the literature by Larson and Story [37] found an association between frequency of snacking and higher energy intake and energy from sugars but not with BMI among adolescents. A meta-analysis found that higher eating frequency in boys (2–19 years) was associated with lower body weight status [38]. Varying definitions and methods of quantifying eating frequency further complicate findings [34].

## 3. Intake during Independent Eating Occasions

Within the home and family environment, changing social and economic factors may contribute to changes in the frequency of independent eating occasions experienced by early adolescents. For example, about 70% of all USA mothers with children under 18 years were working or looking for work in 2014 [39]. This high rate of maternal employment, as well as paternal employment, could result in fewer family meals and older children taking care of themselves with respect to preparing and eating meals. About 20% of teens responding to a national parent/teen survey of lifestyle behaviors indicated they often eat alone [40,41]. A population-based survey of adolescents in one metropolitan area showed that the majority (69%) had assisted with dinner preparation at least once in the past week [42]. Several additional studies have also assessed frequency of involvement in food preparation among older children and adolescents [42,43,44,45,46]. However, these studies did not determine whether the assessment was based on assisting a parent who was present or whether the involvement was associated with an independent eating occasion where older children/adolescents were caring for themselves.

The frequency of family meals has been associated with numerous benefits regarding diet quality [47], which may be attributed to offering meals that include healthy foods and beverages, as well as benefits from having parents encourage and model healthful intake at mealtime [48]. Based on NHANES data 2007–2010, the frequency of the meals the family ate together with a youth between the ages of 6 and 19 years varied from 4.2 to 6.1 meals per week when both race/ethnicity and income were considered [49]. Therefore, children may be eating alone or in locations away from family members in the home for possibly 1–3 meals per week. In a cross-sectional study of approximately 1000 children (10–14 years) in Ontario, the majority (77%) reported consuming dinner with a parent 6–7 nights per week [50], with non-Whites and lower income children reporting less frequent family dinners. In this study, a higher weekly family dinner frequency was associated with fewer calories consumed by children during an afternoon snack, fewer calories consumed by children at the dinner meal, and a higher total number of eating occasions for the day. Eating dinner alone was associated with eating more calories at dinner and fewer total eating occasions per day. Information is limited about how the eating environment (whether the TV is on, location of the meal, how the meal is served) differs when children eat dinner with family members *vs.* eating dinner alone and how the eating environment might affect intake.

Parental influence via parenting practices is based on the parent’s presence and involvement in the daily life and routines of an early adolescent [51]. Parent supervision during eating occasions at home likely varies with child age and autonomy, parent employment/availability, and socio-demographic variables, such as socioeconomic status. Presence and involvement provides an opportunity to implement parenting practices regarding role modeling, encouraging positive behaviors, and modifying availability of foods and beverages in the home environment. Family involvement has been assessed previously in a cross sectional study involving children (10–11 years) including measures of a child’s time spent alone without an adult at home after school, weekday frequency of family meals, and a parent being physically active with a child [52]. When children spent less time alone after school they consumed fewer soft drinks and had less screen time.

Few studies have examined the effect of parental supervision on diet quality of older children and adolescents. One study conducted by Miller *et al.* [53] used activity diaries to document time spent outside of the school day eating unhealthy foods (high-fat snacks, sweets, sugared drinks, high-sodium snacks, and high-calorie/low-nutrient dense food) among normal and overweight middle school children. Most of the time spent eating was supervised by an adult (86%). However, youth reported spending about 19% of their time eating alone. For about two-thirds of the time they ate alone, they were eating unhealthy food.

Eating meals together and involvement in food preparation are positive factors affecting diet quality among children and adolescents. However, limited information is available regarding associations between eating alone or when unsupervised and diet quality and energy balance.

## 4. Increasing Autonomy and Relationship to Food Choices during Independent Eating Occasions

The transition from childhood to adolescence is characterized by marked shifts in cognitive development, including an increased sense of autonomy in decision-making [54,55], enhanced importance of self-identity, and stronger reliance on peers for acceptance [51]. These developmental changes, along with the environmental context in which an adolescent lives (e.g., family dynamics and practices, household economic status, community acceptance of racial/ethnic backgrounds, and internal personality traits) have a profound impact on decision-making and level of engagement in risky health behaviors [54,55,56]. Data from the 2013 USA Youth Risk Behavior Surveillance survey indicate that top contributors to adolescent mortality and morbidity are engagement in high-risk behaviors, such as unhealthy eating and physical activity patterns, as well as unintentional injuries and violence, substance abuse, and sexual activity leading to pregnancy and sexually-transmitted diseases [57].

Research focusing on adolescent eating habits often reports on behaviors during mid- or late-adolescence, noting that these are transitional life stages [58,59,60,61]. While often not included in nutrition-related studies of adolescents, early adolescents (10–14 years) represent an important group to target in examining eating habits. Previous studies have noted that children and early adolescents face particular food- and eating-related issues, such as food restriction and pressure to eat from parents [62,63]. This may result in high levels of disinhibited emotional eating and lead to unhealthy beliefs about food and eating that are transformed into self-imposed rules about what foods are acceptable and how much one should eat [63,64]. Several studies have also identified dieting concerns and behaviors in this population [65,66]. Given the high energy and nutrient intakes needed to support pubertal physical growth during the period of early adolescence and the potential nutrition-related problems in this group, it is crucial to understand early adolescent eating habits both with and without parental supervision.

During early adolescence, family and parents exert a strong protective influence on food choices and eating habits [67,68,69]. However, because of an increasing sense of independence during this transitional life stage and the need for a sense of control over food choice [70,71,72], children begin making more decisions about their own eating patterns (e.g., the timing and location of foods consumed), and the influence of the family and parents begin to diminish [73,74,75] leading to an increase in selection of energy-dense, nutrient-poor foods. NHANES data demonstrated increased energy intake from quick-service restaurants in adolescents compared to children, and a decline in energy intake from the school cafeteria [76]. Similarly, added sugar intake by food purchase location differed in children and adolescents, with a greater percentage of added sugars in the diet of adolescents coming from grocery stores, quick-service and full-service restaurants, and a lesser percentage from school cafeterias [77]. In an examination of dietary sources of energy, solid fat and added sugars among youth in the USA, a number of differences in intake between children and adolescents were identified [78]. Younger children, for example, obtained a greater share of their solid fat from both whole and reduced-fat milk, while 14–18-year-olds obtained a greater share from fried potatoes and beef. Consumption of added sugars from SSBs increased with age, with average daily consumption of 121 kcal for 4–8-year-olds (30 grams sugar), 169 kcal for 9–13-year-olds (42 grams sugar), and 260 kcal for 14–18- year-olds (65 grams sugar) [78]. These findings are noteworthy because food choices during this transitional life stage impact both current health and health status later in life [72]. Information regarding adolescent food intake and purchasing behaviors is essential in addressing current nutrition-related issues in adolescents such as high rates of obesity.

## 5. Socio-Ecological Model—Influence on Food Choice on Independent Eating Occasions

According to the socio-ecological framework, there is a complex interplay of factors at multiple levels of influence that determine an early adolescent’s weight status [79]. With this framework in mind, risk of obesity regarding early adolescent children is influenced by personal factors, one primary influence being eating patterns (*i.e.*, what foods are chosen, where the foods are consumed, who the foods are consumed with, when the foods are consumed). Personal factors are, in turn, influenced by multiple external layers of influence including parents and family (e.g., beliefs and values of the family and parenting style, and practices related to food and eating, such as role modeling and setting rules and expectations) [17,80]; behavioral settings (e.g., foods and beverages available in the home, school, and other community settings) [81,82]; and the social environment (*i.e.*, peer support) [51,83,84]. Some food-related parenting practices need to be implemented in the presence of the early adolescent to be influential (e.g., verbally encouraging intake and role modeling), but others may be effective without the parent being present at the eating occasion (e.g., making healthful foods available, monitoring disappearance of foods). Furthermore, family structure, the role of mothers *vs.* fathers, race/ethnic background, acculturation and socioeconomic status are factors that may mediate the implementation and influence of food-related parenting practices.

## 6. General Parenting, Food-Related Parenting Practices, and Adolescent Eating Behaviors

A number of studies have found an association between general parenting and adolescent eating behaviors. Higher coercive parental control, characterized by intrusive control [85,86]; lower firm control, characterized by lack of consistent limits [87]; and lower parental structuring, characterized by lack of follow through [85], are related to adolescent consumption of increased numbers of unhealthy snacks. Children of emotionally-rejecting parents [88] and overprotective parents [86] eat fewer FV. In contrast, a higher quality of family functioning [89], warm and caring parenting [90], and encouraging and supportive parenting [91] have all been shown to be predictive of healthy adolescent dietary intake.

General parenting style is also predictive of youth eating behaviors. Baumrind [92] initially identified three parenting styles, later characterized by Maccoby and Martin [93] as existing on two dimensions: responsiveness (warmth) and demandingness (control). The three styles are authoritative (high in demandingness and responsiveness), authoritarian (high in demandingness and low in responsiveness), and permissive or indulgent (low in demandingness and high in responsiveness). Maccoby and Martin [93] added a fourth style, which they called neglectful (low in demandingness and low in responsiveness). Kremers *et al.* [94] found that youth of authoritative parents consumed the most fruits followed by children of indulgent parents, with youth of authoritarian and neglectful parents consuming the fewest number of fruits. Most of these studies target adolescent samples and utilize adolescent self-report of eating behaviors. As a result, adolescent reporting on eating behaviors likely includes reference to supervised and unsupervised eating occasions. However, in the design and reporting of these studies, no effort is made to distinguish between the two.

Although research examining the mechanism between general parenting and adolescent eating behaviors is limited, there is evidence that general parenting may exert some of its effects through its impact on child self-awareness, emotion regulation, and behavior regulation. General parenting that is harsh and invalidating leads to poorer child awareness and poorer regulation of emotional arousal [95] and to child and adolescent emotional eating [86,96,97]. Children with poor awareness and poor regulation of emotion have a limited ability to distinguish between negative emotion and sensations of hunger and satiety, and are also more likely to use food in an effort to cope with negative emotions [98,99].

Similarly, adolescents of parents who are high in psychological control [100] and of parents who are indulgent [93,101] have been shown to have lower levels of behavior regulation. Children and adolescents with poor behavior regulation are at increased risk for disinhibited eating [102,103]. Although research has not examined the association between general parenting and emotional and disinhibited eating during independent eating occasions, one might expect these associations to be stronger than in supervised settings. When adolescents are away from supervising adults they are more reliant upon internal controls to regulate intake and are, therefore, more affected by lack of awareness of inner experience, inability to self soothe, and low inhibitory control.

In addition to the direct effects of general parenting on adolescent eating behaviors, there is evidence that general parenting has an impact on adolescent eating behaviors through moderating the association between food-related parenting practices and adolescent eating behaviors. Only a few studies examined the moderating influence of general parenting on the association between food-related parenting practices and adolescent food choice. Restrictive parenting practices were related to decreased adolescent consumption of SSBs when adolescents perceived their parents as being moderately strict and highly involved in their lives [104]. Similarly, Rodenburg *et al.* [105] found that restrictive food parenting was associated with decreased child consumption of SSBs when parents were low on behavior control. Rodenburg *et al.* [105] examined the potential moderating role of general behavioral and psychological control on a number of additional food parenting practices and child eating behaviors. Parental purchasing of fewer unhealthy foods was associated with lower child unhealthy snacking when psychological control was low, parents rewarding children with food was predictive of decreased child fruit intake when behavioral control was high, and parents’ use of food to try to calm child negative emotion was predictive of child unhealthy snacking when parents were high in psychological control.

These studies demonstrate that the general climate of the parent–child relationship, as influenced by general parenting behaviors and styles, impacts how children and adolescents will respond to specific food parenting behaviors. When parents utilize a high level of control, particularly psychological control, and have low involvement in their children’s lives, their specific food-related parenting practices are less effective. A study by Rodenburg *et al.* [88] is somewhat contradictory to these findings. Parent fruit consumption was most predictive of increased child fruit consumption when parents exhibited high psychological and behavioral control. It is important to note that the fruit consumption data were obtained from parents, and as such likely reflect child fruit consumption in the presence of the parent. It is probable that these findings would be different if examined during independent eating occasions, as research indicates that children of highly controlling parents are less likely to comply with parental expectations when unsupervised by their parents [106]. Research on general parenting indicates that in addition to influencing how responsive children are to parent wishes and expectations, the quality of general parenting influences how likely children are to internalize parental values and to be influenced by those values when unsupervised by their parents [107]. Therefore, research examining the association of these variables during independent eating occasions will be important in advancing this literature.

The influence of family structure is also an important factor to consider. A small body of research has demonstrated a connection between family structure and adolescent eating behaviors. Adolescents from single-parent households, relative to adolescents from dual-parent households, eat less fruits and dairy products [108], are less likely to regularly eat breakfast [109,110], and consume higher amounts of dietary fat [111]. Stewart and Menning [112] found that adolescents from single-parent, step-parent, or no-parent households were less likely to eat vegetables, consumed more fast food, and were more likely to skip meals. This may be at least partly related to less parental monitoring of meals. Non-resident father involvement was related to increased vegetable consumption and decreased skipped meals [112], providing further support for parental monitoring. Single parents tend to have lower incomes, experience higher levels of stress, and spend less time in direct caregiving roles [113]. Therefore, in single-parent households there may be multiple pathways of influence on adolescent independent eating behaviors including decreased availability of healthy food choices, increased independent eating occasions as a result of fewer family meals, and less parental socialization regarding making healthy food choices. Furthermore, socio-economic disadvantage and high stress levels are predictive of harsher and less nurturing general parenting behaviors [114], which may influence adolescent independent eating through its effects on adolescent self-regulation as described above.

Previous studies of the influence of parenting practices on early adolescent eating behaviors have primarily addressed maternal influences. However, a recent review examined the role of fathers [115]. Most studies reported on feeding practices of very young children with few focusing on fathers’ roles with respect to older children and adolescents. For those involving older children and adolescents (6–15 years), fathers were more likely to exert greater pressure to eat than mothers [116,117,118]. Fathers who were more concerned about their child’s weight were more likely to have a higher level of restriction [119]. Finally, youth BMI was positively associated with greater attempts by fathers to promote healthy eating behaviors [120]. None of these studies addressed the influence of fathers on early adolescent eating behaviors during independent eating occasions.

## 7. Modeling

Cruwysa *et al.* [121] reported a robust social modeling effect for eating behavior among 64 experimental studies (published from 1974 to 2014), despite variability in methodological approach, food type, eating context, demographic characteristics of participants, and presence of the role model. These findings indicate that social modeling is an important influence on food intake and largely for food choice. Intake modeling may be reduced when certainty is increased; established routines and preferences can somewhat decrease the modeling effect. Social modeling is not influenced by hunger, age, restraint or weight and is strongest among snack consumption and within peer groups. Social norms establish cues that provide a guide to appropriate action, particularly when there is uncertainty about the behavior and/or a shared identity with the norm or peer group. Social norms may affect food choice and intake by altering intake perceptions and/or the sensory acceptability evaluation of foods [122]. There is much evidence that social norms about eating have a powerful effect on both food choice and amounts consumed.

Modeling is strongest when there is need for affiliation (or desire for such), and perceived and actual similarity with the model. Modeling is a form of behavioral mimicry that occurs often without conscious awareness [121]. Parents are considered to be the most important influencers regarding children’s energy-balance related habits. Palfreyman *et al.* [123] developed and used a measure to investigate associations between parental modeling with healthy and unhealthy food intake between mothers and their children (18 months to 8 years). This self-report measure identified verbal modeling; unintentional modeling (children have eating behaviors that parents had not actively modeled); and behavioral consequences (children’s eating behaviors directly associated with parental modeling). Maternally perceived consequences of behavioral modeling were related to increased FV intake in both mothers and children whereas unintentional modeling was related to higher levels of savory snack intake in the mother/child dyads [123]. These findings are consistent with those of Brown and Ogden [124] showing significant correlations between unhealthy snack foods consumed the previous day by children (9–13 years) and parents. Another study showed that parental influence was more important than peers with respect to adolescent FV intake, with descriptive norms (modeling or what they do) being stronger influencers than what they said or told their children to do (injunctive norms) [125]. Both family (parents) and peers serve as social models for eating [121]. Social modeling of eating occurs when the behavior of others guides what and how much one chooses to eat [121].

However, when children become older and gain more behavioral autonomy, parental influence may become less important than that of peers [126,127]. A recent review confirmed the influence of friends on eating and activity behavior—adolescents eat more healthy foods and are more active when peers eat more healthy foods and are more active [128]. Previous research has found associations between SSB consumption and fast-food restaurant visits among adolescents and their friends [129]. Di Noia and Cullen [130] found that among low-income youth, FV consumption attitudes, descriptive norms, and normative peer behavior predicted perceived but not actual intake.

Peers may have greater influence upon behavior when they are perceived to be more similar to oneself or when there is a perceived shared group membership. “Out-groups” or those with perceived failure to act within social norms are usually not modeled [121]. Social judgment appears to be a concern related to eating behavior [122]. Among 10–12 year old subjects, peer and parental influence were related both to perceived norms and modeling of behaviors (eating breakfast, drinking soda, TV viewing, physical activity) [126]. Youth reported more unfavorable modeling of their peers for TV viewing and consuming soda and more favorable modeling of their parents for breakfast consumption and physical activity. When examined across various healthy behavior practices, a weaker association to friends’ unfavorable behaviors and modeling was found when parental rules for such behaviors were in place.

While social influence of parents and peers is important, little work has specifically examined intake among adolescents eating alone or during independent eating occasions. Several studies have shown that college students ate more when given information about how much another subject ate [131]. Others have shown that intake matching occurred when only information or a video of peer intake was provided and peers were not physically present [132]. Overweight children ate more when eating alone, and friends had greater influence than unfamiliar peers [133,134].

## 8. Availability

Food availability has a great impact on adolescent food choice [51], which is reflected in the literature examining the connection between the two [15]. A systematic review of observational studies was conducted to examine environmental correlates of energy, fat, FV, snack/fast food and soft drink intakes in children and adolescents [15]. A number of studies demonstrated a positive association between availability of specific foods in the home and adolescent intake.

Availability of FV in the home and the potential impact on intake of these foods has been studied extensively. For example, in a sample of 4th–6th graders and their parents, availability in the home was significantly related to intake of FV and 100% fruit juice [135]. In another study involving a survey of adolescents and their parents, availability of FV in the home and a more-healthful/less-healthful food ratio were significantly associated with FV intake [136]. Of note, home availability of FV was the determinant of FV intake among children and adolescents that was the best supported by research evidence [137].

The availability of less nutritious foods in the home has also been associated with undesirable food choices. Pearson *et al.* [138] found that home availability of energy-dense snack foods was positively associated with consumption of these foods among adolescent boys and girls. Similarly, Hispanic early adolescents who had SSBs available at home had lower diet quality than those who did not [139]. Qualitative studies also reflect the impact of availability of less healthful foods on intake. In a study of overweight/obese adolescents, participants described availability of less healthful foods as barriers to healthful eating, highlighting the importance of targeting the home food environment to enable healthier food choices [140].

As the link between home availability of various types of food and intake has been highlighted in a number of studies, this is an important factor to consider when examining influences on choices during independent eating occasions. The presence of both healthy and unhealthy options in the home may impact foods consumed in this setting when adolescents are left to make their own decisions about what to eat and drink.

## 9. Rules and Expectations

In the literature on parenting styles, food rules, and healthy eating among adolescents, parents often adopt two different general food rules. These are (1) food restriction and (2) pressure-to-eat in response to concerns about their child’s weight and dietary intake patterns [85,141]. Parents may pressure their children to eat certain healthy foods, such as FV and restrict intake of unhealthy foods [142,143,144]. Parents may use a combination of these food rules to obtain desired children’s eating patterns [16,17]. In a qualitative study involving parents of early adolescents [145], parents indicated they often managed expectations for sweetened beverage intake by limiting the availability and accessibility at home or by only offering or allowing specific beverages to be consumed.

Birch and colleagues [146] have proposed that food restriction is likely to promote a negative impact on children’s future dietary intake. When the restriction is removed, restricted unhealthy foods become more favored and in turn children over-consume these foods [146]. Alternatively, an “appropriate division of responsibility” is often proposed as a more appropriate approach to food-related parenting [143,147,148]. In this approach, the parent controls which foods are made available and accessible and offered to the child, who in turn decides whether and how much to eat.

Although the use of food-related parenting practices has been supported in nutrition education, evidence of the association between parenting rules and adolescents’ eating is limited and remains equivocal with studies reporting no effect among older children [149] or no longitudinal association [150]. Additionally, studies on food-related parenting practices conducted across socio-demographic characteristic of parents of adolescents are limited. Loth *et al.* [151] examined how parents of adolescents differ for food-related parenting practices across socio-demographic characteristics, including parent gender, race, education level, and household income. High levels of both food restriction and pressure-to-eat coexisted. This pattern was most common among minority and low-income parents. The close examination of the use of particular feeding strategies among these low-income parents identified the concern about their adolescent child not eating enough food. It was also common for these parents to require their child to eat all of the food on their plate at mealtimes or to encourage their child to keep eating even when they were not hungry. Similar findings were reported among low-income mothers who face economic hardship and constant or periodic food insecurity [16]. Parents may feel added pressure to encourage food consumption when food is sufficient and may restrict access to certain unhealthy foods to ensure that their child eats foods of higher nutritional quality [152,153,154]. In another study, adolescents from low-income homes had less supportive home meal environments, fewer eating rules and poorer home availability of FV than adolescents from high-income homes [155].

Studies on family rules and their influence on promoting healthy eating in children have been reported frequently, however, some aspects need further research. These include the influence of family rules in diverse populations where culturally appropriate rules are examined for a variety of foods, family rules for foods consumed away from home or outside of family meals, and independent eating occasions.

## 10. Influence of Race/Ethnicity and Acculturation on Implementation of Food-Related Parenting Practices

Implementation of food-related parenting practices during occasions when parents are present and during independent eating occasions may be reliant on parent and household demographic factors including race/ethnicity and degree of acculturation. For example, the availability of healthy or unhealthy foods in the home could vary based on diet transitions to more Western foods and fewer traditional foods after immigration [156,157]. Analysis of food sources of calcium showed differences among foreign-born *versus* USA-born Asian or Hispanic parents and early adolescent children [158]. These findings indicate that some families may prefer to retain aspects of their culture-of-origin identity, with implications for implementation of food-related parenting practices. Several studies have found differences in home availability of healthful and less healthful foods between race/ethnic groups [49,159]. However, few studies have examined differences in frequency of other parenting practices such as role modeling or setting expectations/rules [160,161] and none during independent eating occasions.

## 11. Call for Research to Inform Obesity Prevention Interventions Focusing on Independent Eating Occasions

First, studies are needed that determine the contribution that foods and beverages consumed during independent eating occasions make to overall diet quality and total daily energy intake among early adolescents. These studies could also better characterize how often early adolescents experience independent eating occasions, as well as provide information about contextual factors, such as location, time of day, activities that occur while eating, and presence of peers or siblings. This information may be helpful in determining how these factors affect intake. This information could also be used to design interventions to promote intake of healthful foods and beverages and limit intake of those that are less healthful.

Studies are also needed that determine how parenting practices affect intake during independent eating occasions. For example, information about how home availability of foods and beverages affects intake may be helpful with respect to interventions that promote redesigning pantries or kitchens to promote intake of healthful snacks and beverages. Information about whether parental rules and expectations for intake are influential when parents are not present would allow parents to clarify expectations or modify rules to improve effectiveness. Studies are also needed that determine the ability of parents to act as a role model to influence what children eat when they are not around. A limited set of parenting practices have been examined in this review, however, other food-related parenting practices that fit the Socio-Ecological model have also been described, such as discussing, educating, involving, and using encouragement [162]. These practices would also need to be examined for effectiveness during independent eating occasions among early adolescents. The literature regarding food intake and influence of parenting practices is primarily based on eating occasions in general. This review calls for more research on targeted eating occasions that occur when parents and caregivers are not present. These studies can determine the importance of independent occasions to overall diet quality, prevalence of obesity, and health of early adolescents. Finally, research is also needed that examines the relationships between parent and family socio-demographic factors such as income, race/ethnicity, acculturation, and sex of the parent and early adolescent, and influence of parenting practices on eating behaviors during independent eating occasions.
